# The thistle study: a stepped-wedge clustered trial of an intrapartum emergencies training package in Scottish maternity units

**DOI:** 10.1186/1745-6215-14-S1-P142

**Published:** 2013-11-29

**Authors:** Erik Lenguerrand, Graeme MacLennan, Dimitrios Siassakos, Timothy Draycott, Siladitya Bhattacharya, John Norrie

**Affiliations:** 1 University of Bristol, Bristol, UK; 2 University of Aberdeen, Aberdeen, UK

## Background

The Stepped-Wedge clustered RCT(SW-cRCT) design is often proposed, but less often executed. It appears to offer significant advantages for intervention where on balance the outcome is likely to be beneficial. It pragmatically implements the intervention for all sites, reduces the logistical/financial challenges of implementation at scale, whilst providing a robust framework for the study of effect trends.

This presentation describes the protocol of the Thistle study, a SW-cRCT evaluating the effectiveness of a training aiming to improve perinatal care and outcomes in term babies across Scotland.

## Study

This study will be conducted between September 2013 and February 2016 for 12 maternity units, and will have five steps, each step lasting six months. Four units per step will be randomized to the intervention at one of the three possible randomisation steps. Each unit will be followed for a minimum of 12 months to investigate effect trends (between 2 to 4 follow-up steps).

## Intervention

*PROMPT*, a multiprofessional team training for maternity staff (http://www.promptmaternity.org).

## Data

Routinely collected information from the Scottish Morbidity Record(SMR02) submitted by maternity hospitals will be interrogated.

## Statistical analysis

The prevalence of babies born with a low Apgar score will be modelled using generalized linear mixed models to investigate the intervention effect, taking appropriate account of the clustering (babies within maternity units). Secondary analyses will adjust for time intervals (steps modelled as fixed effects) and potential interactions between time and treatment effects.

## Conclusion

This presentation aims to provide a methodological insight of a SW-cRCT to share experience about a type of trial design under-used and under-documented.

